# P-375. Long-Term Suppressive Antimicrobial Therapy in Prosthetic Vascular Graft Infection: a Five-Year Retrospective Evaluation

**DOI:** 10.1093/ofid/ofae631.576

**Published:** 2025-01-29

**Authors:** Alessandra Imeneo, Grazia Alessio, Ludovica Ferrari, Tiziana Mulas, Iannetta Marco, Andrea Ascoli Marchetti, Arnaldo Ippoliti, Loredana Sarmati

**Affiliations:** Tor Vergata University of Rome, Rome, Lazio, Italy; Tor Vergata Hospital, Rome, Lazio, Italy; Tor Vergata Hospital, Rome, Lazio, Italy; Tor Vergata, Rome, Lazio, Italy; University of Rome Tor Vergata , Rome, Lazio, Italy; Tor Vergata, Rome, Lazio, Italy; Tor Vergata, Rome, Lazio, Italy; Tor Vergata University, Rome, Rome, Lazio, Italy

## Abstract

**Background:**

Prosthetic vascular graft infection (PVGI) is a rare but life-threatening complication of surgery. Complete removal and replacement of the endograft is the only curative strategy but is frequently impracticable for the high mortality risk, therefore, suppressive antimicrobial therapy (SAT) is the best treatment option.

This retrospective study aimed to describe the indication and outcome of SAT in patients with a PVGI evaluated since 2019 at Tor Vergata Hospital in Rome.

**Figure:**
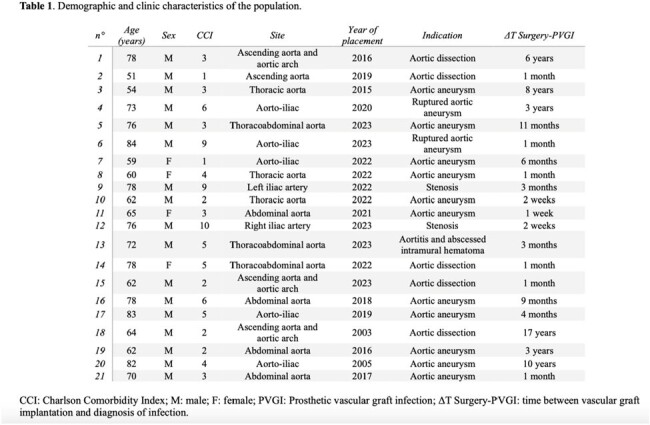

**Methods:**

Patients with a PVGI (no cardiac valve replacement infections) were enrolled. The outcome was defined based on the latest available outpatient evaluation. PVGI was stratified as early/late and suspected/diagnosed according to guidelines.

**Figure:**
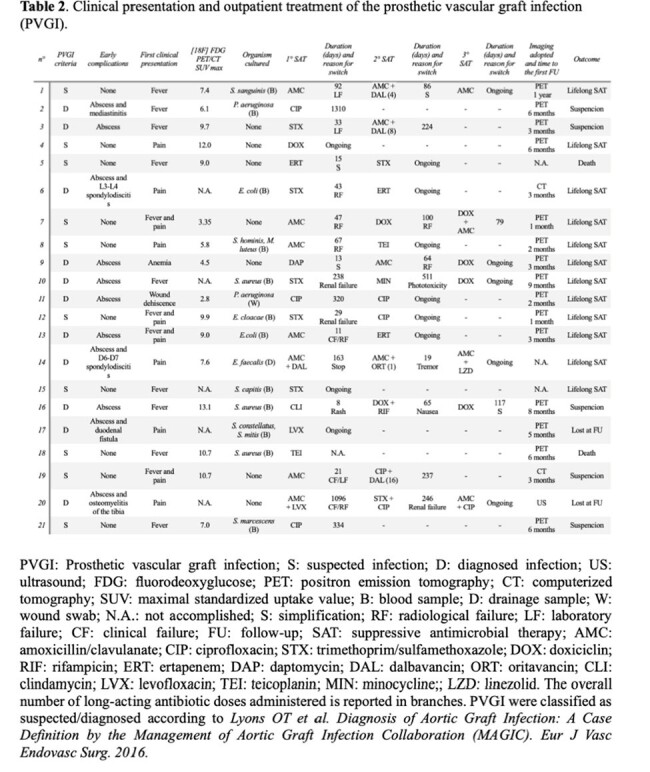

**Results:**

Twenty-one patients were enrolled (Tab. 1-3). At least one causative organism was identified in 41% of cases, mainly through blood samples (57%): 38% were Gram positive (GPB) and 29% were Gram negative (GNB) bacteria. Unexpectedly, early PVGI were more frequently caused by GNB than GPB (p-value 0.006).

All patients were SAT treated; only 3 patients underwent surgery without reaching source-control. Several options, including long-acting antibiotics (LAA), were available for GPB, while cotrimoxazole, ciprofloxacin and ertapenem were the principal long-term SAT for GNB.

The CT-PET scan monitoring was done after a median follow-up (FU) time of 90 [IQR 90-180] days; a positive early CT-PET FU (≤3 months from the first test) was associated with treatment failure needing a modification of SAT (p-value 0.003). Drug adverse effects occurred in 38% of cases and were followed by a change in SAT.

Subsequently to clinical/radiological improvement (71%) or patient decision (29%), SAT was discontinued in 7 patients after a median time of 336 [292-404] days. Patients who experienced SAT discontinuation were younger, with fewer comorbidities, early infections, known pathogen and LAA therapy, although no statistically significant association was observed. Two patients showed a radiological failure needing to resume treatment; 3 patients (14%) successfully suspended SAT currently without relapse.

**Figure:**
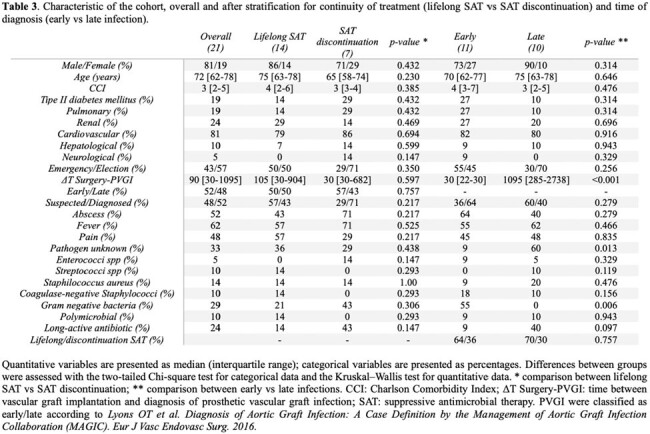

**Conclusion:**

Our results support SAT as a suitable option for patients with PVGI at high surgical risk. Larger prospective studies are needed to guide empirical practice.

**Disclosures:**

**All Authors**: No reported disclosures

